# Revisiting conventional noncovalent interactions towards a complete understanding: from tetrel to pnicogen, chalcogen, and halogen bond†

**DOI:** 10.1039/d3ra06078k

**Published:** 2023-10-27

**Authors:** Cam-Tu Phan Dang, Nguyen Minh Tam, Thanh-Nam Huynh, Nguyen Tien Trung

**Affiliations:** a Faculty of Natural Sciences, Duy Tan University Da Nang 550000 Vietnam phandcamtu@duytan.edu.vn; b Institute of Research and Development, Duy Tan University Da Nang 550000 Vietnam; c Faculty of Basic Sciences, University of Phan Thiet 225 Nguyen Thong Phan Thiet City Binh Thuan Vietnam; d Institute of Catalysis Research and Technology, Karlsruhe Institute of Technology Eggenstein-Leopoldshafen 76344 Germany; e Laboratory of Computational Chemistry and Modelling (LCCM), Quy Nhon University Quy Nhon City 590000 Vietnam

## Abstract

Typical noncovalent interactions, including tetrel (TtB), pnicogen (PniB), chalcogen (ChalB), and halogen bonds (HalB), were systematically re-investigated by modeling the N⋯Z interactions (Z = Si, P, S, Cl) between NH_3_ – as a nucleophilic, and SiF_4_, PF_3_, SF_2,_ and ClF – as electrophilic components, employing highly reliable *ab initio* methods. The characteristics of N⋯Z interactions when Z goes from Si to Cl, were examined through their changes in stability, vibrational spectroscopy, electron density, and natural orbital analyses. The binding energies of these complexes at CCSD(T)/CBS indicate that NH_3_ tends to hold tightly most with ClF (−34.7 kJ mol^−1^) and SiF_4_ (−23.7 kJ mol^−1^) to form N⋯Cl HalB and N⋯Si TtB, respectively. Remarkably, the interaction energies obtained from various approaches imply that the strength of these noncovalent interactions follows the order: N⋯Si TtB > N⋯Cl HalB > N⋯S ChalB > N⋯P PniB, that differs the order of their corresponding complex stability. The conventional N⋯Z noncovalent interactions are characterized by the local vibrational frequencies of 351, 126, 167, and 261 cm^−1^ for TtB, PniB, ChalB, and HalB, respectively. The SAPT2+(3)dMP2 calculations demonstrate that the primary force controlling their strength retains the electrostatic term. Accompanied by the stronger strength of N⋯Si TtB and N⋯Cl HalB, the AIM and NBO results state that they are partly covalent in nature with amounts of 18.57% and 27.53%, respectively. Among various analysis approaches, the force constant of the local N⋯Z stretching vibration is shown to be most accurate in describing the noncovalent interactions.

## Abstract 2

The conventional noncovalent interactions are revisited and systematically investigated using different analysis approaches to offer a step forward in the complete understanding of their nature and behaviors.

## Graphical abstract

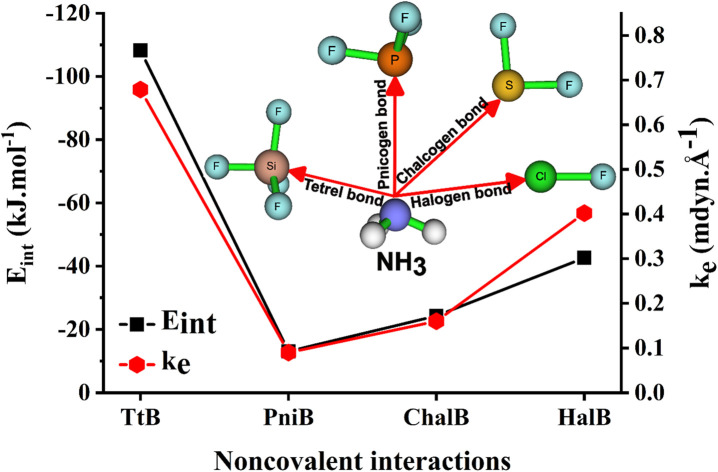

## 1. Introduction

Understanding noncovalent interactions is an essential issue due to their central role in supramolecular materials. Scientists have now oriented these weak intermolecular interactions in designing crystals and metal-containing compounds,^1,2^ but they remain lacking experimental determinations of the interactions' characteristics themself. Tetrel (TtB), pnicogen (PniB), chalcogen (ChalB), and halogen (HalB) bonds are among typical noncovalent interactions that have been extensively investigated in recent years.^3–9^ These interactions' families are generally characterized by the connection between a nucleophilic center Z, usually involving a specific molecule, and an electrophilic center, which could be an electron-rich region or a Lewis base. Alternatively, they can be described in other manners, *i.e.*, σ-hole interactions and charge transfer.

The first works of the conventional NH_3_–SiF_4_ TtB model were explored in the 1980s and mainly focused on its stable structures.^10–12^ Chehayber *et al.* claimed three stable geometrical types of this dimer using *ab initio* MO calculations at STO-6G basis set, encompassing axial, equatorial, and square pyramidal structures.^11^ The NH_3_–SiF_4_ TtB complex was reported to have some degree of covalency in the N–Si bond^11^ but still be driven by electrostatic term.^10,11^ Numerous studies on the electronic properties and other factors influencing its strength have been carried out in recent years.^13–17^ In addition, a systematic work on TH_*n*_F_4−*n*_–NH_3_ complexes elucidated the factors affecting the TtB in detail was reported by Scheiner in 2017.^3^ The pnicogen family was first discovered in the work of Solimannejad *et al.*^18^ and rapidly attracted the interest of the scientific community because of its similarities with the early-known noncovalent bonds, *i.e.*, hydrogen bond (HB) and HalB. It was found that trihalogenphosphines PX_3_ (X = F, Cl, Br) and R_1_R_2_Y molecules (Y = O, S, Se) were able to engage with nitrogen bases NCH and NH_3_ to form different noncovalent interactions, but the global minimum structures were stabilized by N⋯P PniB and N⋯Y ChalB (Y = O, S, Se).^19–21^ Previous theoretical and experimental studies on the H_3_N–ClF heterodimer indicated that primary bonded interaction in its equilibrium structure belongs to the N⋯F HalB.^22–25^ Recently, Chandra *et al.* successfully demonstrated the superior strength of Cl–P⋯N phosphorus bond, overcoming the H–N⋯Cl in NH_3_–PCl_3_ complex using matrix isolation infrared spectroscopy and theoretical calculations.^20^ Comparative strengths of TtB, PniB, ChalB, and HalB and contributing factors were assessed using *ab initio* calculations with the representation of third-row atoms (Ge, As, Se, and Br; respectively).^14^ However, for typical noncovalent interactions relating second-row atoms (Si, P, S, and Cl), to the best of our knowledge, there is no study systematically investigating their changes in geometrical structures, stability, and characteristics when going from TtB to HalB.

Different approaches could be, and often are, simultaneously utilized to scrutinize the characteristics of the interactions in question. These methods are diverse in the descriptors used for characterizing as well as in the underlying theoretical backgrounds. Elgengehi *et al.* stated the good prediction in calculating interaction energies of noncovalent interactions can be made by high-order SAPT with the MP2 correlation and dispersion correction.^26^ Within the scheme of energy decomposition analysis, it was shown that the electrostatic force predominantly stabilizes the noncovalent interactions besides the supplementary contributions from other elements, *i.e.*, dispersion and induction. Besides, the spectroscopic data could provide a reliable description of the intrinsic strength of these interactions.^27–33^ This is critical because one can predict the strength of interaction of interest from information obtained either computationally through quantum mechanical calculations or experimentally *via* vibrational spectroscopies, given good descriptors are determined. In fact, local vibrational mode analysis, originally introduced by Konkoli and Cremer, is known as an *in situ* measure of bond strength.^34,64^ This has been employed in a series of works to assess the strength of noncovalent bonds quantitatively.^13,28,32,35^ Additionally, the chemical bonding within noncovalent complexes could be investigated by electron-density-based methods, such as Atom in Molecule (AIM) and NCI analysis.^36,37^ The utilization of such a variety of methods for studying noncovalently bonded systems often raises the question of which descriptors are better at representing the interaction. In this context, it is necessary to conduct an investigation in which the characteristics of different types of weak interactions are elucidated systematically by different methods.

This work was performed to simulate these noncovalent interactions in a highly systematic manner with the aim of thoroughly discovering the electronic structures, and stability of NH_3_–ZF_*n*_ complexes (Z = Si, P, S, Cl; *n* = 1–4), as well as providing a fundamental understanding of various interactions including TtB, PniB, ChalB, and HalB. The characteristics and relative strength of these interactions can be rationalized by utilizing different modern approaches to investigate the chemical bonding, *i.e.*, NCIplot, high-order SAPT, local vibrational force constant, and also, the conventional methods of electron analyses (AIM and NBO). We highlight the agreements and disagreements between different approaches and provide corresponding explanations where possible. Ultimately, we propose the descriptors that best represent all the interactions of interest.

## 2. Computational details

The geometries of complexes and monomers were optimized at the MP2/aug-cc-pVTZ and B3LYP-D3/def2-TZVP level of theory. The B3LYP-D3 has been claimed to sufficiently describe the binding energy of noncovalent interactions,^39^ while the TZ basis set by Ahlrichs and coworkers (def2-TZVP)^65^ is recommended to give good results.^38^ The harmonic vibrational frequencies were then calculated at the same level to confirm if the structure is truly a minimum on the potential surface and as well as to obtain the zero-point energy (ZPE). Intrinsic features of a chemical bond, including changes in bond length, vibrational mode, and force constant, were analyzed to observe the effects of intermolecular interactions on involved covalent bonds in isolated monomers and to determine the characteristics of intermolecular interactions. Specifically, the local stretching force constant derived from the local vibrational mode analysis theory was calculated to describe the strength of noncovalent interactions by using the LmodeA-nano code as a Pymol plugin.^40,41^

The values of binding energy (*E*_b_) were calculated with optimized complexes and separately optimized monomers based on the supermolecular method. In addition, to measure the actual interaction occurring within a complex formation, the interaction energies (*E*_int_) were determined by the energetic difference between the complexes and the isolated monomers adopted from the corresponding complexes. These energetic quantitative terms were corrected for the basis set superposition error (BSSE) using the counterpoise procedure^42^ and ZPE. The extrapolation to the complete basis set (CBS) was based on the focal-point method, using the Helgaker equation: *E*(*ζ*) = *E*_CBS_ + *a* exp(−*bζ*).^43^ In particular, the MP2/CBS electronic energies were extrapolated from the single point calculations at MP2 and aug-cc-pVTZ, aug-cc-pVQZ, and aug-cc-pV5Z basis sets (abbreviated as aTZ, aQZ, a5Z). The ΔCCSD(T) correction term was calculated at the aTZ basis set.

The Atoms in Molecules (AIM) theory of Bader *et al.*^36^ was used to analyze properties at the bond critical point (BCP) of the interactions formed and provide the topological graphs. These calculations were done at the MP2 density employing the AIMall program.^44^ The noncovalent interaction regions were quantitatively determined with the NCIplot 4.2 ^63^ from the MP2/aDZ density at MP2/aTZ geometries. The 2D and 3D visualization of these interactions were plotted by means of Gnuplot^45^ and VMD software.^46^ The calculations of Natural Bond Orbital (NBO)^47^ was performed at ωB97X-D/aTZ level to elucidate the intermolecular interactions between NH_3_ and fluoride compounds (SiF_4_, PF_3_, SF_2_, ClF). The Natural Resonance Theory (NRT) was also applied to obtain their bond orders and partitioning into covalent and ionic contributions. Molecular electrostatic potentials (MEP) were computed at MP2/aTZ and analyzed on the 0.001 a.u electron density surfaces with the GaussView package. The location and values of maxima (*V*_s,max_) and minima (*V*_s,min_) of electrostatic potential were derived using Multiwfn.^48–50^ All quantum chemical calculations were carried out using Gaussian16 rev.03 program.^51^

The calculations of high-order SAPT2+(3) with MP2 correlated correction^52^ were performed at the aTZ basis set *via* the PSI4 program to evaluate the contribution of different physical terms and the interaction energies of studied complexes.

## 3. Results and discussion

### 3.1. Geometrical structures, molecular electrostatic potential and stability

**Fig. 1:**
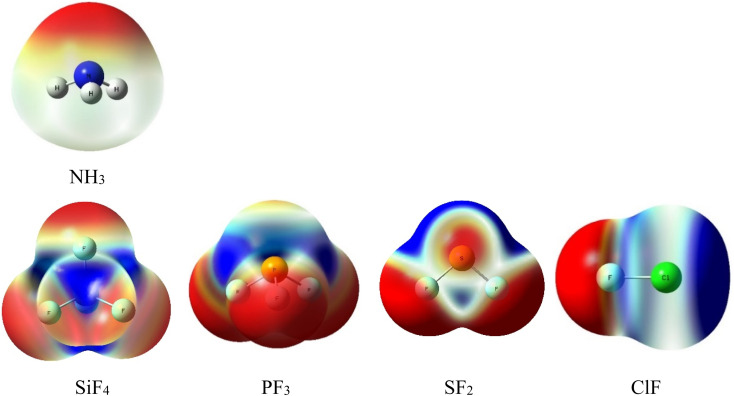
Electrostatic potential surfaces of isolated monomers computed at MP2/aTZ (isovalue density = 0.001 a.u).

The initial prediction of intermolecular interactions formed in complexes of NH_3_ with ClF, SF_2_, PF_3_, and SiF_4_ is based on the MEP surfaces at 0.001 e bohr^−3^ contours with the densities computed at MP2/aTZ (Fig. 1). The color ranging from red to blue indicates different values of potential which change from the most negative to the most positive, respectively. The *V*_s,min_ and *V*_s,max_ extrema values corresponding to the stationary points of these MEP surfaces are collected in Table S1 of the ESI.†

The lone pair of N corresponds to the intense red region of NH_3_. This interactive site favors connecting with the blue part (positive potential) of SiF_4_, PF_3_, SF_2_, and ClF. The numbers of σ-holes characterizing these molecules are observed to decrease from 4 to 1, corresponding to the number of F atoms in fluoride compounds. According to Fig. 1, all σ-holes lie on the extension of the Z–F bonds (Z = Si, P, S, Cl). The MEP reproduction generally agrees well with the previous studies.^3,10,15,16,53,54^ Remarkably, we found fully three σ-holes of PF_3_ in this work, whereas Alkorta *et al.* reported only two maxima of PF_3_ but three for PCl_3_ and PBr_3_ molecules at MP2/aug′-cc-pVTZ level of theory.^19^

Interestingly, the surface minima in ClF anisotropically locate around the extension of the Cl–F bond as a strip and perpendicular to the axis of the Cl–F bond. These minima, however, do not fit the position of the expected σ-hole. This phenomenon results from these minima resonating with each other, which, in turn, creates a σ-hole on the outermost portion along the extension of the Cl–F bond (*ca.* Table S1†).^27,55^

**Fig. 2:**
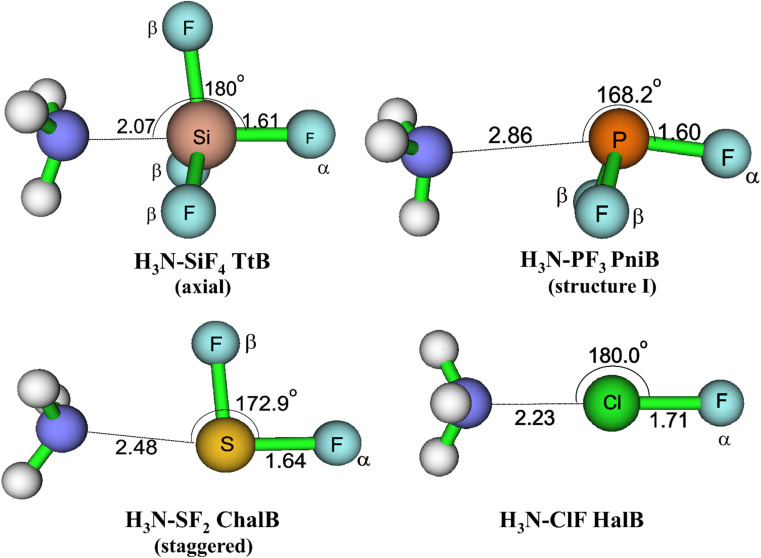
Geometrical structures of intermolecular-bonded complexes of NH_3_ with ClF, SF_2_, PF_3_, and SiF_4_ at MP2/aTZ level.

The equilibrium geometries of targeted complexes at MP2/aTZ, shown in Fig. 2, exhibit only one type of intermolecular interaction: N⋯Si TtB, N⋯P PniB, N⋯S ChalB, and N⋯Cl HalB. These N⋯Z formations (Z = Si, P, S, Cl) are confirmed by the existences of bond critical points (BCPs) and path bonds between N and Z atoms in their topological graphs (Fig. S1†). Other less stable structures of these models are represented in Fig. S2.† The binding energies of investigated complexes are collected in Table 1, in which the performances of B3LYP-D3 method are also assessed.

**Table 1:** Binding energies of noncovalent complexes at various levels of theory (*E*_b_, kJ mol^−1^)^a^

	MP2/aDZ	MP2/aTZ	CCSD(T)/aTZ^b^	CCSD(T)/CBS^c^	B3LYP-D3/aTZ	B3LYP-D3/def2-TZVP
H_3_N–SiF_4_ TtB	−30.3 (−49.8)	−22.3 (−32.6)	−23.8	−23.7	−22.5	−18.3
H_3_N–PF_3_ PniB	−13.4 (−21.2)	−12.3 (−15.7)	−12.8	−12.5	−13.8	−13.5
H_3_N–SF_2_ ChalB	−21.5 (−29.9)	−21.1 (−24.8)	−18.7	−20.4	−25.6	−25.5
H_3_N–ClF HalB	−34.4 (−42.0)	−36.6 (−40.2)	−28.7	−34.7	−47.5	−46.9

a Values in parentheses are *E*_b_ with only ZPE correction.

b The ZPE and BSSE corrections were calculated at MP2/aTZ.

c ZPE corrections were calculated at MP2/aTZ.

For the TtB model, the calculations found three conformers of H_3_N–SiF_4_, including axial (Fig. 2), equatorial, and square structures (Fig. S2†). The H_3_N–SiF_4_ axial geometry has a high symmetry of *C*_3v_ and intermolecular distance between N and Si of 2.07 Å at MP2/aTZ geometry (2.14 Å at B3LYP-D3/def2-TZVP). Three F atoms in the initial equilibrium tetrahedral structure of SiF_4_ rearrange to expand the space for NH_3_ moiety interacting as the fifth ligand. By considering the BSSE contribution to the *E*_b_ value, our work provides highly reliable results of −22.3 kJ mol^−1^ for *E*_b_ of H_3_N–SiF_4_ at MP2/aTZ and −23.7 kJ mol^−1^ at CCSD(T)/CBS. These values are less negative than those obtained at *ab initio* MO calculations combined with STO-6G (−0.433 eV ≈ −41.8 kJ mol^−1^),^11^ at MP2/6-311G(3df,2p)//B3PW91/6-311G(3df,2p) (−9 kcal mol^−1^ ≈ −37.7 kJ mol^−1^),^10^ MP2/aug′-cc-pVTZ (−45 kJ mol^−1^).^15^ However, it is worth noting that our ZPE- and BSSE-corrected *E*_b_ values of NH_3_–SiF_4_ at MP2/aDZ differ from the result reported by Scheiner at the same level of theory, *i.e.*, −30.3 *vs.* −44.3 kJ mol^−1^.^3^ It is obviously due to the lack of ZPE correction in the binding energy calculated in their work. Therefore, we are convinced that our modestly negative *E*_b_ values are more reliable.

Regarding the PniB, the H_3_N–PF_3_ equilibrium geometry obtained associates with the N⋯P bond length of 2.86 Å at MP2/aTZ and agrees well with previous studies.^19,53^ In particular, the lone pair of NH_3_ interacts with one σ-hole in PF_3_, as predicted from MEP analysis. This geometrical structure was also pointed out as the ground state of analogous complexes of PCl_3_ and PBr_3_.^19,20^ The calculated *E*_b_ values in this work are −12.3 kJ mol^−1^ at MP2/aTZ and −12.5 kJ mol^−1^ at CCSD(T)/CBS, which are less negative than that at MP2/aug′-cc-pVTZ (*E*_b_ = −19.8 kJ mol^−1^).^19^ Also, two hydrogen bonded conformers of H_3_N–PF_3_ (structure II and III) are found (Fig. S2†) but considerably less stable than the H_3_N–PF_3_ PniB on the PES.

For the H_3_N–SF_2_ complex, the global minimum on the PES is the staggered structure, in which the SF_2_ molecular plane lies at the staggered position to NH_3_. It is worth mentioning that Otilia Mó *et al.* proposed the eclipsed structure to be the most stable structure of H_3_N–SF_2_.^56^ The re-calculations of two H_3_N–SF_2_ conformers confirm the global minimum of staggered one (*cf.* Fig. S2 of ESI†). The *E*_b_ value of staggered H_3_N–SF_2_ complex at CCSD(T)/CBS is −20.4 kJ mol^−1^, close to the value obtained at MP2/aTZ but less negative than that at B3LYP-D3/def2-TZVP by 5.1 kJ mol^−1^ (Table 1).

The H_3_N–ClF structure characterizes the bond length of N⋯Cl HalB of 2.23 Å and the N⋯Cl–F bond angle of 180.0° at MP2/aTZ. This N⋯Cl bond of H_3_N–ClF was previously reported as a conventional HalB with a negative (*r*_1_–*r*_2_), where *r*_1_ and *r*_2_ are distances of F–Cl and Cl–N, respectively.^25^ The binding energy of H_3_N–ClF is computed at −36.6 kJ mol^−1^ at MP2/aTZ and −34.7 kJ mol^−1^ at CCSD(T)/CBS, surpassing that of the H_3_N–ClF hydrogen-bonded structure (−1.38 kJ mol^−1^ at MP2/aTZ, *cf.* Fig. S2†). This result is in good agreement with the *E*_b_ value calculated at MP2/6-311++G(d,p) by Alkorta *et al.* (−8.86 kcal mol^−1^ ≈ −37.07 kJ mol^−1^).^57^

The binding energies of typical noncovalent complexes in this study were also calculated using B3LYP functional with D3 dispersion correction of Grimme *et al.*^58^ in conjunction with two different basis sets, *i.e.*, Dunning type – aTZ and Ahlrichs type – def2-TZVP. From Table 1, both B3LYP-D3/aTZ and B3LYP-D3/def2-TZVP do not effectively describe the binding energy of these typical complexes with respect to those derived at CCSD(T)/CBS extrapolation. The result thus does not agree with the observation found in CH_2_XOH–CO_2_ (X = F, Cl, Br) complexes in which the B3LYP-D3 showed excellent descriptions of stability.^39^

Incredibly, the characteristic region obtained from MEP surfaces of the involved monomers completely fits the equilibrium structures found at MP2/aTZ. This implies a dominant role of Coulomb electrostatic force upon complexation. Since these complexes share the NH_3_ host molecule, the maxima electrostatic potentials *V*_s,max_ of electrophilic fragments, including SiF_4_, PF_3_, SF_2_, and ClF, are considered (*cf.* Table S1†). Their *V*_s,max_ decreases in the order of SiF_4_ > ClF > SF_2_ > PF_3_. However, the complex stability decreases in the order of H_3_N–ClF HalB > H_3_N–SiF_4_ TtB > H_3_N–SF_2_ ChalB > H_3_N–PF_3_ PniB; this does not follow the decreasing trend of *V*_s,max_ of Z central atoms (Z = Si, P, S, Cl). This inconsistency reveals that the binding energy cannot serve as an adequate descriptor for intermolecular strength. It can be clarified by the calculations of interaction energies and the effect of the deformation energy in the following section, which reflects the energy cost of involved monomers to bind each other and achieve the complex geometry.

### 3.2. Interaction energy analyses and energetic decomposition

The high-order SAPT approach is an effective alternative to estimate the interaction energy (*E*_int_) of intermolecular complexes and decompose it into meaningful physical energy terms. Table 2 provides the *E*_int_ values of studied complexes derived from various orders of SAPT and supermolecular methods.

**Table 2:** Interaction energies obtained from supermolecular and SAPT approaches (kJ mol^−1^)

*E*_int_	H_3_N–SiF_4_ TtB	H_3_N–PF_3_ PniB	H_3_N–SF_2_ ChalB	H_3_N–ClF HalB
MP2/aTZ^a^	−108.3	−13.1	−24.3	−42.6
CCSD(T)/aTZ^a^	−111.4	−13.5	−21.2	−33.1
B3LYP-D3/def2TZVP^a^	−94.3	−14.6	−29.1	−53.5
SAPT0	−175.9	−28.1	−45.4	−63.9
SAPT2	−156.5	−22.0	−37.3	−56.5
SAPT2+	−166.7	−24.3	−39.5	−56.6
SAPT2+(3)	−156.8	−22.3	−35.1	−50.5
SAPT2+dMP2	−135.6	−20.5	−34.9	−53.9
SAPT2+(3)dMP2	−125.6	−18.6	−30.5	−47.8
SAPT2+(3)dMP2^b^	−109.8	−13.7	−22.5	−37.1

a *E*_int_ corrected ZPE and BSSE.

b *E*_int_ derived from SAPT2+(3)dMP2 approach corrected ZPE.

Generally, the accuracy of *E*_int_ according to the SAPT approach tends to improve significantly with the increase of order with respect to that using the supermolecular method at MP2/aTZ. Specifically, a high deviation is found between the interaction energies obtained from CCSD(T)/aTZ and lower order of SAPT calculations, *e.g.*, SAPT0. By increasing the order of perturbation, the SAPT-based interaction energies get closer to those derived from CCSD(T)/aTZ. As a result, the SAPT2+(3)dMP2** approach exhibits an effective description of *E*_int_ of noncovalent complexes (with the highest difference of only 4.0 kJ mol^−1^ in the case of H_3_N–ClF HalB, as compared to *E*_int_ value derived from CCSD(T)/aTZ), and is consistent with the observation of Elgengehi *et al.*^26^

The obtained interaction energies based on the supermolecular methods at three levels of theory, *i.e.*, MP2/aTZ, CCSD(T)/aTZ, and B3LYP-D3/def2-TZVP, confirm again the ineffectiveness of the B3LYP-D3/def2-TZVP in evaluating the typical noncovalent interactions. The interaction energy of the TtB complex is significantly negative, of −111.4 kJ mol^−1^ at CCSD(T)/aTZ and −109.81 kJ mol^−1^ using SAPT2+(3)dMP2** approach while the corresponding *E*_b_ is only −23.8 kJ mol^−1^. This large deviation results from the withdrawing electron effects of three F atoms and the rearrangement of the SiF_4_ geometry, leading to the substantial deformation energy in H_3_N–SiF_4_ TtB complex (∼87.6 kJ mol^−1^) and in good agreement with an amount of 86.9 kJ mol^−1^, reported by Scheiner.^3^ The high strength of N⋯Si TtB might be due to its partially covalent nature and will be clarified in later sections. A similar trend was previously stated in the case of the HF_3_Ge–NH_3_ complex.^14^

Based on Table 2, the bond strength of noncovalent interactions grows in the following order: N⋯P PniB < N⋯S ChalB < N⋯Cl HalB < N⋯Si TtB. This result is in line with the prediction of MEP analysis and differs from the trend obtained from the binding energies (Table 1). Therefore, the interaction energy should be a better bond strength descriptor in comparision with the binding energy.

**Fig. 3:**
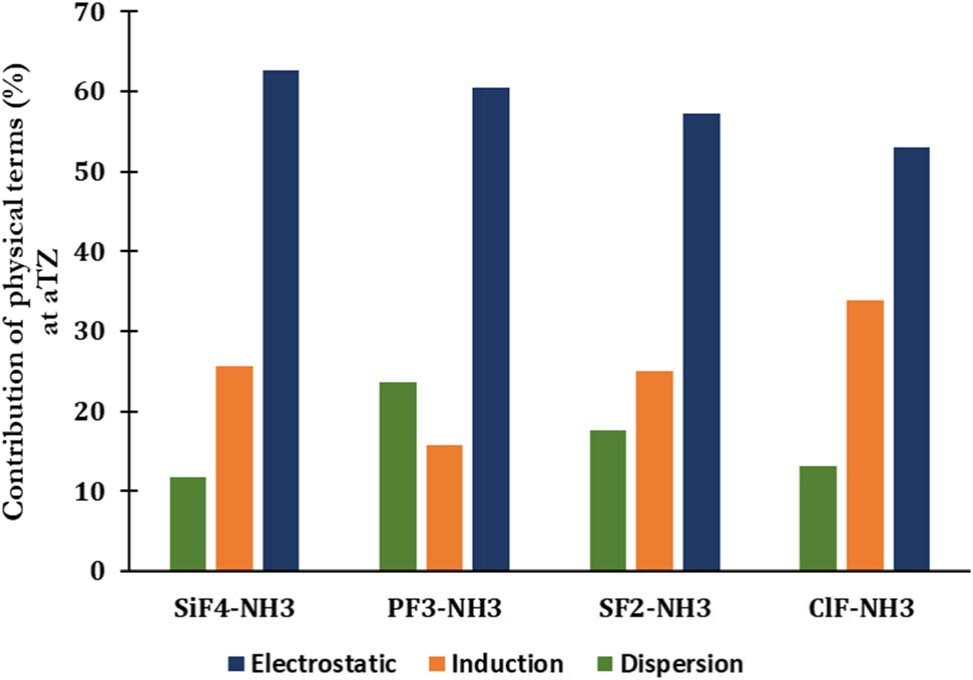
SAPT decomposition of the total interaction energy of complexes between H_3_N with SiF_4_, PF_3_, SF_2_, and ClF at the aTZ basis set.

The percentage of each component contributing to the strengths of noncovalent interactions unveils the nature of noncovalent interactions (Fig. 3 and Table S2 of ESI†). The electrostatic term remains to govern the strength of complexes (53–63%), indicating that it primarily drives the strength of corresponding noncovalent interactions. The contribution of dispersion and induction terms are in ranges of 11–24% and 16–34%, respectively. The relative contribution of the exchange component to the total interaction energy typically increases when going from the complex with ClF to SiF_4_, with the absolute ratio *E*_exch_/*E*_int(SAPT)_ rising from 3.21 to 5.39, respectively (Table S2†).

### 3.3. Vibrational spectroscopy characterizing noncovalent interactions

In addition to the stability and energy decomposition, the intrinsic features of noncovalent interactions are further described through the changes in intermolecular distances and stretching frequencies engaged. The related adjustments of internal geometries, including the selected changes of Z–F_α_, N–H_α_ intramolecular bonds (Δ*r*) and local stretching frequencies (Δ*ν*), local vibrational frequencies of N⋯Z intermolecular interactions (*ν*(N⋯Z)) and the corresponding force constant (*k*) are collected in Table 3.

**Table 3:** Selected structural parameters of complexes optimized at MP2/aTZ^a^

	H_3_N–SiF_4_ TtB	H_3_N–PF_3_ PniB	H_3_N–SF_2_ ChalB	H_3_N–ClF HalB
Δ*r*(Z–F_α_)	38	15	33	76
Δ*r*(N–H_α_)	1.93	1.29	0.83	0.15
Δ*ν*(Z–F_α_)	−101	−47	−113	−216
Δ*ν*(N–H_α_)	12	−6	4	20
*ν*(N⋯Z)	351	126	167	261
*k*(N⋯Z)	0.679	0.009	0.160	0.402

a Bond distances in mÅ, frequencies in cm^−1^, angles in degree, local stretching force constant *k* in mdyn Å^−1^.

The four typical noncovalent interactions are characterized by the elongations of Z–F_α_ (Z = Si, P, S, Cl) and N–H_α_ bond lengths, in ranges of 15–76 mÅ and 0.83–1.93 mÅ, respectively, which initially reveal the weakening of the involving bonds Z–F_α_ and N–H_α_ upon complexation. The Si–F_α_ bond length in H_3_N–SiF_4_ complex is lengthened by an amount of 38 mÅ at MP2/aTZ, as compared to the original one Si–F in SiF_4_ structure. This result is completely in line with the work of Scheiner *et al.* (0.037 Å).^3^ Applied the characteristic proposed by Del Bene *et al.* to the investigated interactions in this work,^59^ we also observed the negative value of (*r*(Z–F_α_) − *r*(N⋯Z)), indicating they are all conventional noncovalent interactions. The stretching frequencies of Z–F_α_ bonds shift to the red region with magnitudes of 47–216 cm^−1^. This finding is typically similar to conventional X–H⋯Y red-shifting hydrogen bonds where X–H and Z–F_α_ both belong to the electrophilic component, while Y and N act as the nucleophilic region.^60,61^ Especially, the N⋯Cl HalB formation encompasses a significant shift of −216 cm^−1^ in *ν*(Cl–F), confirming the strong effect of N⋯Cl HalB formation to the Cl–F covalent bond involved.

The local vibrational force constant is a reliable measure to examine the noncovalent interaction's strength directly, besides other methods of interaction energy. From Table 3, the formation of N⋯Z interactions is accompanied by stretching frequencies *ν*(N⋯Z) of 351, 126, 167, and 261 cm^−1^, with regards to TtB, PniB, ChalB, and HalB, respectively. More importantly, the *k*(N⋯Z) at MP2/aTZ increases in order N⋯P PniB < N⋯S ChalB < N⋯Cl HalB < N⋯Si TtB, confirming the strength order of investigated noncovalent interactions, as concluded from the energetic and MEP analyses.

The *k* of P⋯N PniB associated with the stretching mode at 126 cm^−1^ computed at MP2/aTZ is 0.009 mdyne Å^−1^, significantly smaller than the corresponding value of PniB in NH_3_–PH_2_NO_2_ complex, which was reported to be 0.144 mdyne Å^−1^ (159.3 cm^−1^) at ωB97X-D/aug-cc-pVTZ.^32^ It is relatively consistent due to the remarkably weaker strength of NH_3_–PF_3_ in this work (−16.7 kJ mol^−1^ for *E*_b_ only corrected only BSSE and −12.3 kJ mol^−1^ for *E*_b_ with ZPE + BSSE corrections) compared to that of NH_3_–PH_2_NO_2_ dimer (*ca.* −31.63 kJ mol^−1^, included BSSE but no ZPE correction, at ωB97X-D/aug-cc-pVTZ).

### 3.4. Topography and electron density analysis

Table 4 summarizes some selected parameters at BCPs of these noncovalent interactions with the density obtained at MP2/aDT. The positive ∇^2^(*ρ*(*r*_c_)) values of all the considered interactions indicate they are all closed-shell configurations. The *ρ*(*r*_c_) at BCPs of Si⋯N TtB and Cl⋯N HalB are considerably higher than those of P⋯N PniB and S⋯N ChalB, confirming the higher strength of the formers. It results from little covalent contribution in their nature, which is deduced from the positive ∇^2^(*ρ*(*r*_c_)) combined with the slightly negative *H*(*r*_c_) and moderate *ρ*(*r*_c_).^62^

**Table 4:** Selected parameters at BCPs of typical noncovalent interactions in investigated complexes (density at MP2/aDZ)^a^

Noncovalent interactions	*ρ*(*r*_c_)	∇^2^(*ρ*(*r*_c_))	*H*(*r*_c_)
N⋯Si	58.3	0.199	−0.017
N⋯P	18.5	0.044	0.000
N⋯S	37.3	0.152	−0.001
N⋯Cl	61.4	0.162	−0.007

a *ρ*(*r*_c_) are in ×10^−3^ a.u, ∇^2^(*ρ*(*r*_c_)) and *H*(*r*_c_) are in a.u.

For the NH_3_⋯SiF_4_ TtB complex, the electron density of the N⋯Si TtB is 58.3 × 10^−3^ a.u (see Table 4) at MP2/aDZ, and quite consistent with the past works.^3,15^ A direct connection between N and Si is observed in its topology derived from AIM analysis, confirming the N⋯Si TtB existence, as also found in the SiF_4_–NCLi complex.^17^ Nevertheless, the CF_4_–NCH and SiF_4_–NCH complexes were pointed out to exhibit three F⋯N bonds, instead of the N⋯Si TtB.^17^ The *H*(*r*_c_) at BCP of N⋯Si TtB is −0.017 a.u, implying its covalent character in nature, which is consistent with the suggestion of Marín-Luna *et al.* at MP2/aug′-cc-pVTZ.^15^

The N⋯S ChalB in the staggered conformer embraces a slightly higher electron density than that in eclipsed one by 7.3 × 10^−3^ a.u, demonstrating the better strength of this ChalB in staggered conformer. For the NH_3_⋯FCl complex, the *ρ*(*r*_c_) at BCP of N⋯Cl HalB is even higher than the criteria for noncovalent interactions with partial covalent character, recently suggested by Kumar *et al.*^62^

**Fig. 4:**
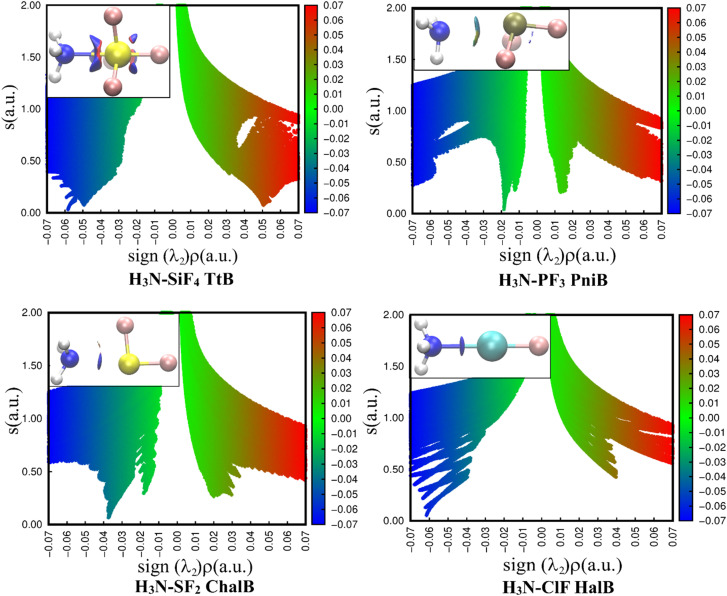
2D scatter plots and 3D isosurface maps of investigated complexes obtained from the NCI analyses (the NCI color scale is −0.07 < *ρ* < 0.07 a.u for SCF densities).

The 2D representations derived from the NCIplot of investigated complexes (Fig. 4) quantitatively reveal different strengths of four noncovalent interactions N⋯Z (Z = Si, P, S, Cl). In all cases, the negative *λ*_2_ displays troughs with electron density ranging from over 0.01 to over 0.06 a.u, suggesting the attractive interactions in these complexes.^37^

A light bluish region was observed over the NH_3_–PF_3_ complex and consistent with the work of Chandra *et al.*^20^ The NCIplot of S⋯N ChalB in NH_3_–SF_2_ is analyzed for the first time in this study. It shows two well-defined troughs in 2D-graph of both NH_3_–PF_3_ and NH_3_–SF_2_. It is strange that AIM did not recognize the smaller interaction rationalizing the small trough. The description of these weak interactions existing in H_3_N–PF_3_ and H_3_N–SF_2_ will be revealed in the NBO analysis section.

### 3.5. Natural bond orbital analysis

We used NBO theory to investigate the formation of noncovalent interactions from the Lewis-like viewpoint. The NBO 7.0 analyses of noncovalent complexes between H_3_N with SiF_4_, PF_3_, SF_2_, and ClF are computed at ωB97X-D/aug-cc-pVTZ level and represented in Table 5. It is observed that an amount of electron transfers from NH_3_ moiety to the remaining component interpreting by the positive charge of NH_3_, in the range of 0.025–0.198*e* upon complexation. In particular, all dimers of NH_3_ and SiF_4_/PF_3_/SF_2_/ClF exhibit the delocalization LP(N) → BD*(X–F) in which an amount of electron transfers from lone pair of N to the anti-bonding orbitals BD*(X–F).

**Table 5:** Selected characteristics derived from NBO 7.0 analysis at ωB97X-D/aug-cc-pVTZ

Complex	Charge NH_3_ (*e*)	Delocalization	*E* ^(2)^ (kJ mol^−1^)	Total/covalent/ionic (% covalent character)
H_3_N–SiF_4_ TtB	0.169	LP(N) → BD*(Si–F_β1_)	83.09	0.4991/0.0927/0.4064 (18.57%)
		LP(N) → BD*(Si–F_α_)	86.44	
		LP(N) → BD*(Si–F_β2_)	83.01	
		LP(N) → BD*(Si–F_β3_)	83.01	
H_3_N–PF_3_ PniB	0.025	LP(N) → BD*(P–F_α_)	17.82	N/A
		LP(N) → BD*(P–F_β1_)	4.90	
		LP(N) → BD*(P–F_β2_)	4.98	
H_3_N–SF_2_ ChalB	0.086	LP(N) → BD*(S–F_α_)	84.18	0.2467/0.0271/0.2196 (10.99%)
		LP(N) → BD*(F_β_–S)	12.51	
H_3_N–ClF HalB	0.198	LP(N) → BD*(F_α_–Cl)	233.17	0.2666/0.0734/0.1933 (27.53%)

The NBO 7.0 results for SiF_4_–NH_3_ in this work identify four delocalization processes LP(N) → BD*(Si–F) whose *E*^(2)^ of each delocalization ∼ 83–86 kcal mol^−1^. Whereas the NBO 3.1 analysis recognizes the SiF_4_–NH_3_ dimer as a single molecule and is consistent with the work of Marín-Luna *et al.*^15^ In such a case, the existence of BD(N⋯Si) bonding orbital indicates that the Si⋯N bond engages in a covalent interaction or has some covalent nature.

The N⋯P PniB of H_3_N⋯PF_3_ complex is characterized by three delocalization steps associated with the electron transfer from LP(N) to BD*(P–F_α_) (in-plane, 17.82 kJ mol^−1^) and two BD*(P–F_β_) (out-plane, ≈4.90 and 4.98 kJ mol^−1^). Two later processes are expected to strengthen further the stability of the complex. However, Alkorta *et al.* stated only one delocalization step LP(N) → BD*(P–F) of 12.9 kJ mol^−1^ at B3LYP/aug′-cc-pVTZ.^19^ The delocalization from the lone pair of nitrogen to σ*(S–F) anti-bonding states an energy of 84.18 kJ mol^−1^. This value is higher than the work of Otilia Mó *et al.* using G4 + MP2 correlation.^56^ In addition, a secondary delocalization LP(N) → BD*(F_β_–S) is found at 12.51 kJ mol^−1^, in consistence with the NCIplot of the second weak interaction found in this complex. Regarding the HalB model, surprisingly, the LP(N) → BD*(F_α_–Cl) governs the NH_3_⋯ClF complex with a large *E*^(2)^ energy of 233.17 kJ mol^−1^, implying the high contribution of covalent character in this kind of interaction.

In addition, the NBO/NRT-based description of noncovalent interactions helps to quantitatively determine the bond order and valency, which is closely related to classical resonance theory concepts. The total bond orders, which include the covalent and ionic contribution of N⋯Z interactions (Z = Si, P, S, Cl), are included in Table 5. As expected, herein the high covalent character of N⋯Si TtB and N⋯Cl HalB is observed, which account for 18.57 and 27.53% of the total values, respectively. Noteworthy, the covalent contribution to total bond order dominantly controls the strength of the corresponding interactions since the covalent percentage varies in the same trend with the interaction stability.

**Fig. 5:**
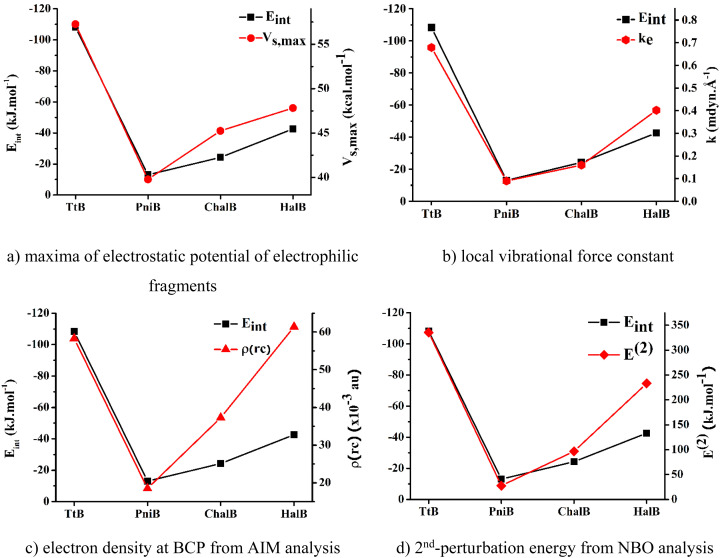
Correlation between the interaction energies and other strength indicators for investigated noncovalent interactions.

To determine the best descriptors of bond strength, we plot the trend derived from different descriptors, namely, electrostatic potentials of isolated nucleophilic fragments, local stretching force constants, electron densities at BCPs, and 2^nd^-perturbation energies against the change in *E*_int_ of the noncovalent interactions of interest (Fig. 5). The local stretching force constant – *k*, is recently recognized as an intrinsic characteristic of molecules that directly and accurately measures the bond strength. Meanwhile, the three remaining properties are traditional indicators utilized to characterize chemical bond strength, yet with some degree of discrepancy. According to Fig. 5, it can be seen that the force constants of the local Z⋯N stretching modes can effectively reproduce the trend obtained from *E*_int_. The electrostatic potential and the 2^nd^ perturbation energy could also qualitatively describe the strength of these weak interactions to a reasonable extent. However, the electron density at BCP derived from Bader's theory shows its poor capacity in presenting the order of interaction strengths. Therefore, it could be concluded that the local stretching force constant is the most accurate descriptor for the strength of these noncovalent interactions.

## 4. Conclusion

In this study, various types of σ-hole interactions are revisited and thoroughly investigated using high-level quantum chemical methods. The binding energies of complexes between NH_3_ and typical Lewis acids (SiF_4_, PF_3_, SF_2_, and ClF) at CCSD(T)/CBS level cover a range of −12.5 to −29.5 kJ mol^−1^. Among these complexes, the NH_3_–ClF HalB dimer was found to be the most stable structure. Notably, the strength order of these interactions deviates from the typical trend when the electron acceptor goes from SiF_4_ to ClF. Instead, the order is as follows: N⋯Si TtB > N⋯Cl HalB > N⋯S ChalB > N⋯P PniB, as derived from the analyses of interaction energies and their local stretching force constant.

Besides the supermolecular approach, the high-order SAPT2+(3)dMP2 accurately calculates the interaction energy and confirms the primary contribution of electrostatic components in stabilizing the complex. For other typical approaches, while the bond strength based on *E*^(2)^ of NBO is effective, the electron density-based ones, *i.e.*, NCIplot and AIM, do not provide accurate results on their stability. The molecular electrostatic potential is recognized as adequate for predicting equilibrium geometries, as well as the strength of noncovalent bonds.

The intrinsic features of noncovalent interactions were also investigated. Upon complexation, the NH_3_–ZF_*n*_ (Z = Si, P, S, Cl; *n* = 1–4) are characterized by the local stretching frequencies of 351, 126, 167, and 261 cm^−1^ at MP2/aTZ level of theory, as Z goes from Si to Cl, respectively. The topological parameters indicate that the investigated N⋯Si TtB and N⋯Cl HalB are partly covalent in nature. The NBO-based NRT analysis confirms this finding, showing that the covalency contributions of these TtB and HalB are 18.57% and 27.53%, respectively, in terms of total bonded order. Finally, among different approaches applied, the force constant of the local N⋯Z stretching vibration is shown to be most accurate descriptor for the strength of noncovalent interactions. The results obtained from this study could provide valuable information for various chemistry disciplines, including supramolecular chemistry, host–guest interactions, as well as crystal engineering, and rational drug design.

## Conflicts of interest

There are no conflicts of interest to declare.

## Supplementary Material

RA-013-D3RA06078K-s001

## References

[cit1] Hajji M., Haouas A., Abad N., Guerfel T. (2023). The Unconventional Noncovalent Interactions Control: Crystallographic and Theoretical Analyses of the Crystalline Structure of 1,1′-(1-Chloro-4-methoxyphenyl)dibenzene as a Case Study. ChemistrySelect.

[cit2] Mahmudov K. T., Kopylovich M. N., Guedes da Silva M. F. C., Pombeiro A. J. L. (2017). Non-covalent interactions in the synthesis of coordination compounds: recent advances. Coord. Chem. Rev..

[cit3] Scheiner S. (2017). Systematic Elucidation of Factors That Influence the Strength of Tetrel Bonds. J. Phys. Chem. A.

[cit4] Scheiner S. (2020). Understanding noncovalent bonds and their controlling forces. J. Chem. Phys..

[cit5] Legon A. C. (2017). Tetrel, pnictogen and chalcogen bonds identified in the gas phase before they had names: a systematic look at non-covalent interactions. Phys. Chem. Chem. Phys..

[cit6] Brammer L. (2017). Halogen bonding, chalcogen bonding, pnictogen bonding, tetrel bonding: origins, current status and discussion. Faraday Discuss..

[cit7] Alkorta I., Elguero J., Frontera A. (2020). Not Only Hydrogen Bonds: Other Noncovalent Interactions. Crystals.

[cit8] Jena S., Dutta J., Tulsiyan K. D., Sahu A. K., Choudhury S. S., Biswal H. S. (2022). Noncovalent interactions in proteins and nucleic acids: beyond hydrogen bonding and π-stacking. Chem. Soc. Rev..

[cit9] Hajji M., Abad N., Habib M. A., Elmgirhi S. M. H., Guerfel T. (2021). Computational chemistry methods for modelling non-covalent interactions and chemical reactivity— an overview. J. Indian Chem. Soc..

[cit10] Politzer P., Murray J. S., Lane P., Concha M. C. (2009). Electrostatically driven complexes of SiF4 with amines. Int. J. Quantum Chem..

[cit11] Chehayber J. M., Nagy S. T., Lin C. S. (1984). *Ab initio* studies of complexes between SiF4 and ammonia. Can. J. Chem..

[cit12] Ault B. S. (1981). Matrix-isolation studies of Lewis acid/base interactions: infrared spectra of the 1:1 adduct SiF4.NH3. Inorg. Chem..

[cit13] Sethio D., Oliveira V., Kraka E. (2018). Quantitative assessment of tetrel bonding utilizing vibrational spectroscopy. Molecules.

[cit14] Dong W., Li Q., Scheiner S. (2018). Comparative Strengths of Tetrel, Pnicogen, Chalcogen, and Halogen Bonds and Contributing Factors. Molecules.

[cit15] Marín-Luna M., Alkorta I., Elguero J. (2017). A theoretical study of the HnF4–nSi:N-base (n = 1–4) tetrel-bonded complexes. Theor. Chem. Acc..

[cit16] Gholipour A. (2018). Mutual interplay between pnicogen–π and tetrel bond in PF 3⊥ X–Pyr⋯SiH3CN complexes: NMR, SAPT, AIM, NBO, and MEP analysis. Struct. Chem..

[cit17] Grabowski S. J. (2014). Tetrel bond–σ-hole bond as a preliminary stage of the SN2 reaction. Phys. Chem. Chem. Phys..

[cit18] Solimannejad M., Gharabaghi M., Scheiner S. (2011). SH⋯N and SH⋯P blue-shifting H-bonds and N⋯P interactions in complexes pairing HSN with amines and phosphines. J. Chem. Phys..

[cit19] Alkorta I., Elguero J., Del Bene J. E. (2015). Exploring the PX3:NCH and PX3:NH3 potential surfaces, with X=F, Cl, and Br. Chem. Phys. Lett..

[cit20] Chandra S., Ramanathan N., Sundararajan K. (2022). Is nitrogen in ammonia an elusive electron acceptingpnicogen in a predominantly phosphorus bonded PCl3:NH3 dimer?. Chem. Phys. Lett..

[cit21] Murray J. S., Lane P., Clark T., Politzer P. (2007). σ-hole bonding: molecules containing group VI atoms. J. Mol. Model..

[cit22] Zou J. W., Lu Y. X., Yu Q. S., Zhang H. X., Jiang Y. J. (2006). Halogen Bonding: An AIM Analysis of the Weak Interactions. Chin. J. Chem..

[cit23] Machara N. P., Ault B. S. (1988). Infrared matrix isolation studies of the 1:1 complexes of chlorine fluoride with nitrogen bases in argon and nitrogen matrices. J. Phys. Chem..

[cit24] Li Q., Lin Q., Li W., Cheng J., Gong B., Sun J. (2008). Cooperativity between the Halogen Bond and the Hydrogen Bond in H3N⋯XY⋯HF Complexes (X, Y=F, Cl, Br). ChemPhysChem.

[cit25] Xu H., Cheng J., Li Q., Li W. (2016). Some measures for making a traditional halogen bond be chlorine-shared or ion-pair one in FCl·NH3 complex. Mol. Phys..

[cit26] Elgengehi S. M., El-Taher S., Ibrahim M. A. A., El-Kelany K. E. (2021). Unexpected favourable noncovalent interaction between chlorine oxyanions (ClOx−; x = 1–4) and benzene: benchmarking DFT and SAPT methods with respect to CCSD(T). Comput. Theor. Chem..

[cit27] Jabłoński M., Palusiak M. (2012). Nature of a Hydride–Halogen Bond. A SAPT-, QTAIM-, and NBO-Based Study. J. Phys. Chem. A.

[cit28] Oliveira V., Kraka E., Cremer D. (2017). Quantitative assessment of halogen bonding utilizing vibrational spectroscopy. Inorg. Chem..

[cit29] Freindorf M., Kraka E., Cremer D. (2012). A comprehensive analysis of hydrogen bond interactions based on local vibrational modes. Int. J. Quantum Chem..

[cit30] Zhang X., Dai H., Yan H., Zou W., Cremer D. (2016). B–H···π Interaction: A New Type of Nonclassical Hydrogen Bonding. J. Am. Chem. Soc..

[cit31] Zou W. and Cremer D., Properties of local vibrational modes: the infrared intensity, in Thom H. Dunning, Jr., A Festschrift from Theoretical Chemistry Accounts, Springer, 2015, pp. 149–163, 10.1007/978-3-662-47051-0_14

[cit32] Setiawan D., Kraka E., Cremer D. (2014). Description of pnicogen bonding with the help of vibrational spectroscopy—the missing link between theory and experiment. Chem. Phys. Lett..

[cit33] Zhao L., Zhi M., Frenking G. (2022). The strength of a chemical bond. Int. J. Quantum Chem..

[cit34] Cremer D., Wu A., Larsson A., Kraka E. (2000). Some Thoughts about Bond Energies, Bond Lengths, and Force Constants. J. Mol. Model..

[cit35] Oliveira V., Cremer D., Kraka E. (2017). The Many Facets of Chalcogen Bonding: Described by Vibrational Spectroscopy. J. Phys. Chem. A.

[cit36] Bader R. F. W. (1991). A quantum theory of molecular structure and its applications. Chem. Rev..

[cit37] Johnson E. R., Keinan S., Mori-Sánchez P., Contreras-García J., Cohen A. J., Yang W. (2010). Revealing Noncovalent Interactions. J. Am. Chem. Soc..

[cit38] Bursch M., Mewes J. M., Hansen A., Grimme S. (2022). Best-Practice DFT Protocols for Basic Molecular Computational Chemistry. Angew. Chem., Int. Ed..

[cit39] Phan Dang C. T., Trung N. T. (2022). Complexes of carbon dioxide with methanol and its monohalogen-substituted: beyond the tetrel bond. Chem. Phys. Lett..

[cit40] Kraka E., Zou W., Tao Y. (2020). Decoding chemical information from vibrational spectroscopy data: local vibrational mode theory. Wiley Interdiscip. Rev.: Comput. Mol. Sci..

[cit41] Tao Y., Zou W., Nanayakkara S., Kraka E. (2022). LModeA-nano: A PyMOL Plugin for Calculating Bond Strength in Solids, Surfaces, and Molecules *via* Local Vibrational Mode Analysis. J. Chem. Theory Comput..

[cit42] Boys S. F., Bernardi F. (1970). The calculation of small molecular interactions by the differences of separate total energies. Some procedures with reduced errors. Mol. Phys..

[cit43] Halkier A., Helgaker T., Jørgensen P., et al (1998). *.*, Basis-set convergence in correlated calculations on Ne, N2, and H2O. Chem. Phys. Lett..

[cit44] Keith T. A. , AIMAll (Version 19.10. 12), TK Gristmill Softw., Overl. Park KS USA, 2019, published online

[cit45] Racine J. (2006). gnuplot 4.0: a portable interactive plotting utility. J. Appl. Econom..

[cit46] Humphrey W., Dalke A., Schulten K. (1996). VMD: Visual molecular dynamics. J. Mol. Graphics.

[cit47] Reed A. E., Curtiss L. A., Weinhold F. (1988). Intermolecular interactions from a natural bond orbital, donor-acceptor viewpoint. Chem. Rev..

[cit48] Zhang J., Lu T. (2021). Efficient evaluation of electrostatic potential with computerized optimized code. Phys. Chem. Chem. Phys..

[cit49] Lu T., Chen F. (2012). Quantitative analysis of molecular surface based on improved marching tetrahedra algorithm. J. Mol. Graphics Modell..

[cit50] Lu T., Chen F. (2012). Multiwfn: a multifunctional wavefunction analyzer. J. Comput. Chem..

[cit51] Frisch M. J. , Trucks G. W. and Schlegel H. B., et al., Gaussian 16 Rev. A. 03, Gaussian Inc., Wallingford CT, 2016

[cit52] Hohenstein E. G., Sherrill C. D. (2010). Density fitting of intramonomer correlation effects in symmetry-adapted perturbation theory. J. Chem. Phys..

[cit53] Wysokiński R., Zierkiewicz W., Michalczyk M., Scheiner S. (2020). How Many Pnicogen Bonds can be Formed to a Central Atom Simultaneously?. J. Phys. Chem. A.

[cit54] Scheiner S. (2018). Comparison of Various Means of Evaluating Molecular Electrostatic Potentials for Noncovalent Interactions. J. Comput. Chem..

[cit55] Eskandari K., Zariny H. (2010). Halogen bonding: a lump–hole interaction. Chem. Phys. Lett..

[cit56] Mó O., Montero-Campillo M. M., Alkorta I., Elguero J., Yáñez M. (2019). Ternary Complexes Stabilized by Chalcogen and Alkaline-Earth Bonds: Crucial Role of Cooperativity and Secondary Noncovalent Interactions. Chem.–Eur. J..

[cit57] Alkorta I., Rozas I., Elguero J. (1998). Charge-Transfer Complexes between Dihalogen Compounds and Electron Donors. J. Phys. Chem. A.

[cit58] Grimme S. (2011). Density functional theory with London dispersion corrections. Wiley Interdiscip. Rev.: Comput. Mol. Sci..

[cit59] Del Bene J. E., Alkorta I., Elguero J. (2010). Do traditional, chlorine-shared, and ion-pair halogen bonds exist? An *ab initio* investigation of FCl: CNX complexes. J. Phys. Chem. A.

[cit60] Chang X., Zhang Y., Weng X., Su P., Wu W., Mo Y. (2016). Red-Shifting *versus* Blue-Shifting Hydrogen Bonds: Perspective from *Ab Initio* Valence Bond Theory. J. Phys. Chem. A.

[cit61] Joseph J., Jemmis E. D. (2007). Red-, Blue-, or No-Shift in Hydrogen Bonds: A Unified Explanation. J. Am. Chem. Soc..

[cit62] Kumar N., Saha S., Sastry G. N. (2021). Towards developing a criterion to characterize non-covalent bonds: a quantum mechanical study. Phys. Chem. Chem. Phys..

[cit63] Boto R. A., Peccati F., Laplaza R., Quan C., Carbone A., Piquemal J.-P., Maday Y., Contreras-García J. (2020). NCIPLOT4: Fast, Robust, and Quantitative Analysis of Noncovalent Interactions. J. Chem. Theory Comput..

[cit64] Konkoli Z., Cremer D. (1998). A new way of analyzing vibrationalspectra. I. Derivation of adiabatic internal modes. Int. J. Quantum Chem..

[cit65] Weigend F., Ahlrichs R. (2005). Balanced basis sets of split valence, triple zeta valence and quadruple zeta valence quality for H to Rn: Design and assessment of accuracy. Phys. Chem. Chem. Phys..

