# Supplementary material for: Revisiting conventional noncovalent interactions towards a complete understanding: from tetrel to pnicogen, chalcogen, and halogen bond
Source: RSC Adv. 2023 Oct 27;13(45):31507–17. doi: 10.1039/d3ra06078k (PMC10606978; doi:10.1039/d3ra06078k)
Supplement: RA-013-D3RA06078K-s001 [file RA-013-D3RA06078K-s001.pdf]

Electronic Supplementary Material (ESI) for RSC Advances.
This journal is © The Royal Society of Chemistry 2023

Supporting information

Revisiting conventional noncovalent interactions towards a complete understanding: From tetrel to pnictogen, chalcogen, and halogen bond

Cam-Tu Phan Dang[1,2]*, Nguyen Minh Tam[3], Thanh-Nam Huynh[4], Nguyen Tien Trung[5]

[1]*Faculty of Natural Sciences, Duy Tan University, Da Nang 550000, Vietnam*

[2]*Institute of Research and Development, Duy Tan University, Da Nang 550000, Vietnam*

[3]*Faculty of Basic Sciences, University of Phan Thiet, 225 Nguyen Thong, Phan Thiet City, Binh Thuan, Viet Nam*

[4]*Institute of Catalysis Research and Technology, Karlsruhe Institute of Technology, Eggenstein-Leopoldshafen 76344 Germany*

[5]*Laboratory of Computational Chemistry and Modelling (LCCM), Quy Nhon University, Quy Nhon City 590000, Vietnam*

**Corresponding author:* phandcamtu@duytan.edu.vn / phancamtu9982@gmail.com.vn

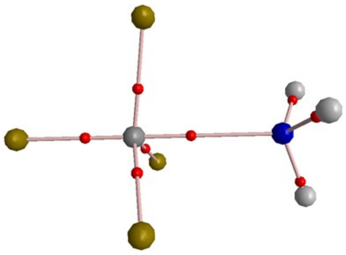

H_3N-SiF_4 TtB

(axial)

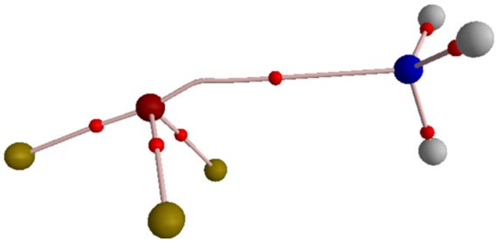

H_3N-PF_3 PniB

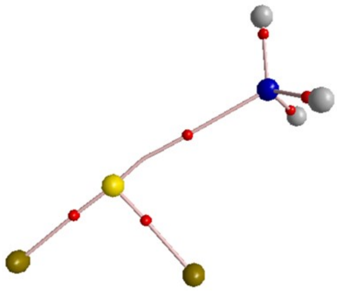

H_3N-SF_2 ChalB

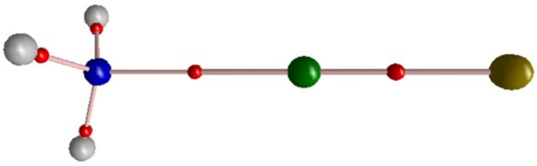

H_3N-ClF HalB

Figure S1. Topological graphs with density at MP2/aDZ of investigated complexes

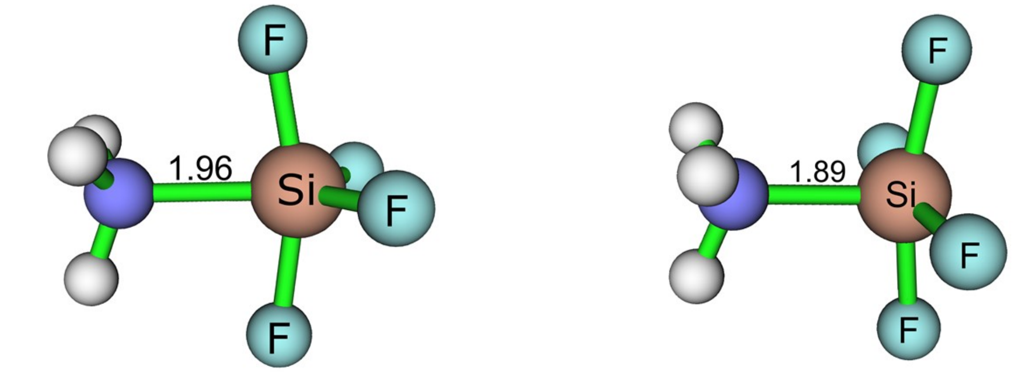

Equatorial **conformer** ***Square pyramidal*** **conformer**

a) Less stable conformers of H_3N-SiF_4 complex at MP2/aTZ

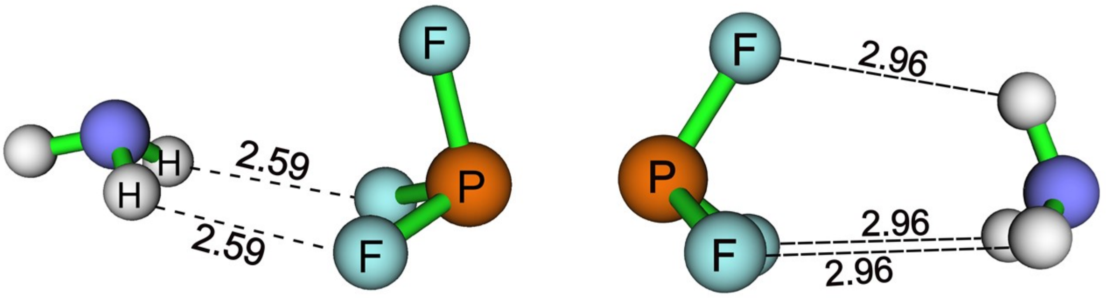

Conformer II **Conformer III**

b) Less stable hydrogen-bonded conformers of H_3N-PF_3 complex at MP2/aTZ

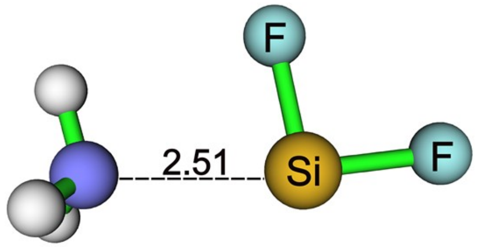

c) ***Eclipsed*** **conformer of H_3N-SF_2**

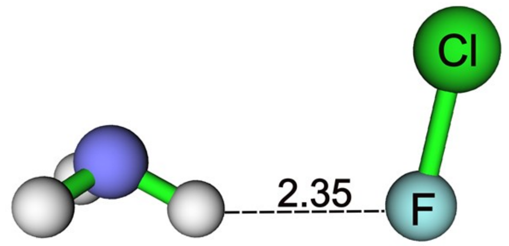

d) Hydrogen bonded-structure of H_3N-ClF

Figure S2. Secondary structures of investigated complexes at MP2/aTZ level

Two other conformers of H_3N-SiF_4, including *equatorial* and *square pyramidal* geometries, stated by Chehayber *et al.*[1], were also reoptimized at MP2/aTZ high-level of theory to accurately investigate their stabilities and electronic properties (cf. Figure S2). Their binding energies are positive, 5.10 and 11.92 kJ.mol^{-1}, respectively, at MP2/aTZ. Both *equatorial* and *square pyramidal conformer* occur an imaginary frequency of -43.14 cm^{-1} and -125.83 cm^{-1}.

The *hydrogen-bonded* conformers (**II** and **III**) of NH_3-PF_3 both are less stable than H_3N-PF_3 PniB one. Indeed, their E_b+ZPE+BSSE values at MP2/aTZ are slightly negative, i.e. -0.56 and -1.64 kJ.mol^{-1}, respectively. The AIM analyses show the conformer **II** exhibits three intermolecular interactions, including a PF···N HalB and two NH···F hydrogen bonds. Their ρ(rc) values at BCPs are 1.9×10^{-3} a.u, 5.8×10^{-3} a.u, and 5.8×10^{-3} a.u, respectively; and all significantly lower than that of N···P PniB (18.5×10^{-3} a.u). The conformer **III** occurs two imaginary frequencies (-49.49 and -49.47 cm^{-1}) at MP2/aTZ.

The *eclipsed* structure of H_3N-SF_2 model exhibits the stabilization energy of -21.2 kJ.mol^{-1} at MP2/aTZ, reasonably approximate to that of the *staggered* one (-21.1 kJ.mol^{-1}). However, one imaginary frequency is found in the *eclipsed* conformer (-57.15 cm^{-1}), where H_α atom of NH_3 vibrates towards the outside of the C_s symmetry plane. Thus, the *eclipsed* structure could be recognized as a transition state moving towards the more stable *staggered* conformer.

The H_3N-ClF *hydrogen-bonded* structure exhibits an binding energy of -1.38 kJ.mol^{-1} at MP2/aTZ.

1. Chehayber JM, Nagy ST, Lin CS. Ab initio studies of complexes between SiF4 and ammonia. *Can J Chem*. 1984;62(1):27-31. doi:10.1139/v84-006

Table S1. $V_{s,min}$ **and** $V_{s,max}$ **values (kcal.mol**$^{-1}$**) of isolated monomers derived from Multiwfn at MP2/aTZ**

Isolated monomers	$V_{s,min}$	$V_{s,max}$
NH_3	-37.267252	26.530416
		26.516305
		26.524880
ClF*	-10.899116	47.815127
SF_2	-11.484665	45.237921
	-11.467289	45.232004
PF_3	-11.311841	39.768458
	-11.267542	39.654171
	-11.269485	39.613856
SiF_4	-9.805767	57.264031
	-9.776024	57.161980
	-9.804366	56.949983
	-9.788675	57.243163

* This structure exists maxima points around the F atom, which form a ring in perpendicular to the F-Cl bond

Table S2. Contribution of different components to interaction energies of noncovalent complexes using high order SAPT2+(3)dMP2 approach ($kJ.mol^{-1}$) at aTZ

	SiF_4-NH_3 TtB	PF_3-NH_3 PniB	SF_2-NH_3 ChalB	ClF-NH_3 HalB
Electrostatic	-330.66	-50.89	-103.60	-162.05
Induction	-135.79	-13.27	-45.35	-103.41
Dispersion	-62.00	-19.89	-31.90	-40.28
Exchange	402.81	65.49	150.34	257.93
E_{elst}/E_{int}(SAPT)	3.39	3.40	2.74	2.63
E_{ind}/E_{int}(SAPT)	2.16	1.49	0.71	1.08
E_{disp}/E_{int}(SAPT)	0.84	1.05	1.07	0.49
E_{exch}/E_{int}(SAPT)	-5.39	-4.93	-3.53	-3.21
